# Loss of Cbl and Cbl-b ubiquitin ligases abrogates hematopoietic stem cell quiescence and sensitizes leukemic disease to chemotherapy

**DOI:** 10.18632/oncotarget.3403

**Published:** 2015-03-19

**Authors:** Wei An, Scott A. Nadeau, Bhopal C. Mohapatra, Dan Feng, Neha Zutshi, Matthew D. Storck, Priyanka Arya, James E. Talmadge, Jane L. Meza, Vimla Band, Hamid Band

**Affiliations:** ^1^ Eppley Institute for Research in Cancer and Allied Diseases, University of Nebraska Medical Center, Omaha, NE 68198, USA; ^2^ Departments of Genetics, Cell Biology & Anatomy, University of Nebraska Medical Center, Omaha, NE 68198, USA; ^3^ Departments of Biochemistry & Molecular Biology, University of Nebraska Medical Center, Omaha, NE 68198, USA; ^4^ Departments of Pathology & Microbiology, College of Medicine, University of Nebraska Medical Center, Omaha, NE 68198, USA; ^5^ College of Public Health, University of Nebraska Medical Center, Omaha, NE 68198, USA; ^6^ Fred & Pamela Buffet Cancer Center, University of Nebraska Medical Center, Omaha, NE 68198, USA

**Keywords:** Cbl, ubiquitin ligase, HSC, quiescence, tyrosine kinase

## Abstract

Cbl and Cbl-b are tyrosine kinase-directed RING finger type ubiquitin ligases (E3s) that negatively regulate cellular activation pathways. E3 activity-disrupting human Cbl mutations are associated with myeloproliferative disorders (MPD) that are reproduced in mice with Cbl RING finger mutant knock-in or hematopoietic Cbl and Cbl-b double knockout. However, the role of Cbl proteins in hematopoietic stem cell (HSC) homeostasis, especially in the context of MPD is unclear. Here we demonstrate that HSC expansion and MPD development upon combined Cbl and Cbl-b deletion are dependent on HSCs. Cell cycle analysis demonstrated that DKO HSCs exhibit reduced quiescence associated with compromised reconstitution ability and propensity to undergo exhaustion. We show that sustained c-Kit and FLT3 signaling in DKO HSCs promotes loss of colony-forming potential, and c-Kit or FLT3 inhibition *in vitro* protects HSCs from exhaustion. *In vivo*, treatment with 5-fluorouracil hastens DKO HSC exhaustion and protects mice from death due to MPD. Our data reveal a novel and leukemia therapy-relevant role of Cbl and Cbl-b in the maintenance of HSC quiescence and protection against exhaustion, through negative regulation of tyrosine kinase-coupled receptor signaling.

## INTRODUCTION

Maintenance of a small pool of hematopoietic stem cells (HSCs) in a quiescence state assures life-long hematopoiesis [1]. Inability to maintain quiescence leads to HSC exhaustion, seen as loss of the long-term reconstitution ability. While our understanding of the mechanisms that enforce and maintain HSC quiescence is incomplete, a number of critical growth factors elaborated by niche components and their specific receptors on HSCs exemplify HSC quiescence regulation through integration of cell extrinsic and intrinsic mechanisms [1].

Many of these growth factors activate receptor tyrosine kinases (RTKs) or protein tyrosine kinase (PTK)-associated receptors, such as stem cell factor (SCF)/c-KIT, FMS-related tyrosine kinase 3 ligand (FLT3L)/FLT3; angiopoietin-1/TIE-2; and thrombopoietin (TPO)/c-MPL [1, 2]. Genetic evidence supports their requirement for HSC quiescence and maintenance [3–6]. Notably, activating mutations of c-KIT, FLT3 or c-MPL, or their downstream PTKs (e.g., JAK2), promote leukemogenesis [7–10]. Yet, mechanisms that dictate the physiological HSC quiescence versus proliferation outcomes downstream of these receptors are undefined.

The Casitas B-lineage Lymphoma (Cbl) family RING finger ubiquitin ligases (E3s) are recruited to a number of RTKs upon their activation and facilitate their turnover [11, 12]. Phosphorylated Cbl proteins also associate with signaling intermediates, e.g., PI3 kinase, Crk and Vav, to further negatively regulate the level and/or duration of activated receptor signaling [12]. Mammals express three Cbl family members: Cbl and Cbl-b are ubiquitously expressed but enriched in hematopoietic cells, while Cbl-c expression is restricted to epithelial cells. The evolutionarily-conserved N-terminal tyrosine kinase-binding (TKB) domain, linker helical region and RING finger domain, found in all three members, form the basic PTK-directed E3 module [12].

Recently, missense mutations or small deletions of Cbl have been identified in about 3–5% of all myeloid malignancies and about 10% of juvenile myelomonocytic and chronic myelomonocytic leukemias [13–16]. Cbl mutations are clustered in the linker helical region and RING finger domain [17, 18], impair the E3 function [13, 19, 20], and are commonly duplicated through acquired uniparental disomy [14, 15, 17]. While the exact mechanism by which mutant Cbl induces leukemogenesis in unclear, a prevailing hypothesis is that of dominant-negative inhibition, in which the mutant protein competes with Cbl-b and the remaining wild type Cbl in rare heterozygous mutant patients, thereby creating a complete Cbl/Cbl-b null state in leukemic cells [13, 14, 21].

Association of Cbl mutations with leukemias suggests an important physiological role of Cbl proteins in HSCs. Studies in animal models also support this possibility. Cbl knock-out (Cbl-KO) mice exhibit expansion of HSCs and multipotent progenitors (MPPs) in the bone marrow (BM) [13, 22] but no frank leukemia. Knock-in mice with E3-inactivating Cbl RING finger mutation (C379A) [23] on a Cbl-null background (referred to as Cbl RF-KI) develop slow but severe MPD, with an average lifespan of a year [24]. Cbl-b-KO mice develop normally but their mature lymphoid cells exhibit hyper-responsiveness to antigenic challenges [25–27].

Early embryonic lethality of whole body Cbl/Cbl-b double knockout (DKO) mice [28] suggested that Cbl and Cbl-b function redundantly in certain biological processes. We showed that MMTV-driven Cre expression in Cbl-flox/flox, Cbl-b-null mice produced a Cbl/Cbl-b DKO in hematopoietic cell lineages (referred to as Cbl/Cbl-b DKO, or DKO), and these mice developed an expanded HSC pool and severe MPD with an average lifespan of only eight weeks [21]. *Ex vivo* analyses showed that BM-derived hematopoietic precursors from Cbl-KO [22], Cbl RF-KI [24] and Cbl/Cbl-b DKO mice [21] exhibit hyper-proliferation in response to cytokines, including SCF, FLT3 and TPO. Crossing of Cbl RF-KI mice with Flt3L-null mice substantially ameliorated the MPD [24, 29]. Interestingly, deletion of Cbl enhanced leukemogenesis in a BCR-Abl transgenic model of chronic myeloid leukemia [13], a disease thought to originate from HSCs [30]. Thus, several lines of evidence support the idea of Cbl proteins as potential brakes in growth factor-induced proliferation of HSCs. How such anti-proliferative role of Cbl-family proteins is integrated into HSC homeostasis remains unknown.

Here, we provide evidence for a previously unknown requirement of Cbl and Cbl-b in the maintenance quiescence and long-term repopulating ability of HSCs. Mechanistically, Cbl and Cbl-b promote HSC maintenance by negative regulation of c-KIT and FLT3. Loss of quiescence due to deletion of Cbl and Cbl-b makes HSCs susceptible to elimination with anti-mitotic therapy. Our studies identify a basic switch that allows tyrosine kinase-coupled receptors on HSCs to maintain quiescence and self-renewal, and suggest that this switch could be therapeutically exploited.

## RESULTS

### Cbl and Cbl-b are redundant but essential regulators of hematopoietic stem and progenitor compartments

Although we have demonstrated that hematopoietic Cbl/Cbl-b deletion promotes MPD, a systematic analysis of DKO vs. single KO mice in HSC regulation was not carried out [21]. Therefore, to firmly establish that Cbl and Cbl-b are redundant in the regulation of HSCs, we examined the occurrence of lethal MPD in 8-week old mice of the following strains: Cbl-KO [31], Cbl-b-KO [25], hematopoietic Cbl/Cbl-b-DKO (MMTV-Cre-driven Cbl deletion on a Cbl-b null background [11, 21], and wild type (WT; control) (genotypes in Supplementary Table 1). Based on peripheral blood (PB) observations of increased total white cell counts, monocytes and granulocytes, extra-medullary hematopoiesis and death of animals (Figure S1A–S1C), development of MPD required the deletion of both alleles of Cbl and Cbl-b. Analysis of BM hematopoietic compartments demonstrated that expansion of HSCs and specific progenitor compartments, including common myeloid progenitors (CMP), granulocyte/macrophage progenitors (GMP) and common lymphoid progenitor (CLP) but not megakaryocyte/erythrocyte progenitors (MEP), was only seen in DKO mice (Figure S1D). Lack of MPD in Cbl^flox/flox^, Cbl-b^WT/del^, MMTV-Cre mice (referred to as Cre control) excluded any MMTV-Cre transgene effects. Quantitative real-time PCR (qPCR) helped establish the complete deletion of Cbl and Cbl-b in HSCs of DKO mice, but not that of other controls (Figure S1E and S1F).

### Cbl and Cbl-b function as intrinsic regulators of HSCs

**Figure 1:**
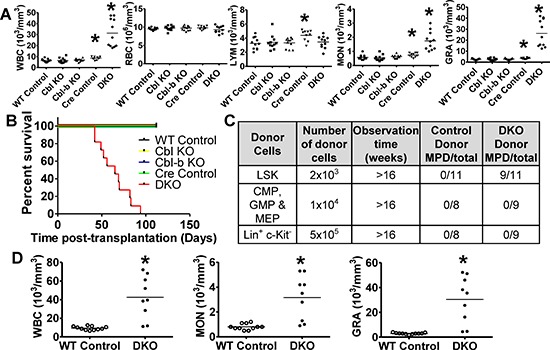
MPD in Cbl/Cbl-b DKO mice is cell autonomous and only LSK cells initiate disease **(A–B)** Whole BM cells were transplanted into lethally-irradiated syngeneic WT recipients; PB counts performed after 4 weeks (A) and survival scored up to 16 weeks (B). Data from two experiments are pooled. Dots represent individual mice. **(C–D)** FACS-sorted subpopulations from WT or Cbl/Cbl-b DKO BM were transplanted with helper cells. (C) MPD (granulocyte count > 10^4^/ml or death before the study was terminated at 16-weeks) after transplantation. (D) PB cell counts at 16-weeks. Data from two experiments are pooled (*p < 0.05).

While transfer of MPD by DKO BM transplant [21] supports a BM cell-intrinsic role of Cbl proteins, the precise disease-initiating cell remains unknown. BM transplants (CD45.2+ donor to CD45.1+ lethally-irradiated syngeneic recipients) confirmed that DKO BM but not the Cbl-KO, Cbl-b-KO or Cre Control BM transplant produced leukocytosis and myelomonocytosis (Figure 1A) with rapid-onset lethality (Figure 1B) in recipients assessed at 4 weeks post-transplant. We next transplanted FACS-sorted subpopulations from WT/DKO BM cells to determine disease initiating cells. Notably, transplant of DKO HSC-enriched LSKs but not that of a pool of CMP, GMP & MEP myeloid progenitors, or Lin^+^ c-kit^−^ mature hematopoietic cells, led to features of MPD, identified by GRA counts > In mRNA level, p57 is more enriched 10^4^/mm^3^ (at 16 weeks) or early death (Figure 1C). Leukocytosis in DKO LSK (Figure 1D) and DKO BM cell recipients (Figure 1A) was comparable. Comparable *in vitro* proliferation of FACS-sorted LSK cells from WT or DKO mice when cultured with DKO or control mouse sera added to growth media (Figure S2) suggests that the phenotypes are unlikely due to factors released by non-hematopoietic tissues impinging on DKO LSKs. Overall, these results demonstrate an HSC cell-intrinsic role of Cbl and Cbl-b whose abrogation allows DKO HSCs to initiate MPD.

### Reduced quiescent HSC fraction in Cbl/Cbl-b DKO mice

**Figure 2:**
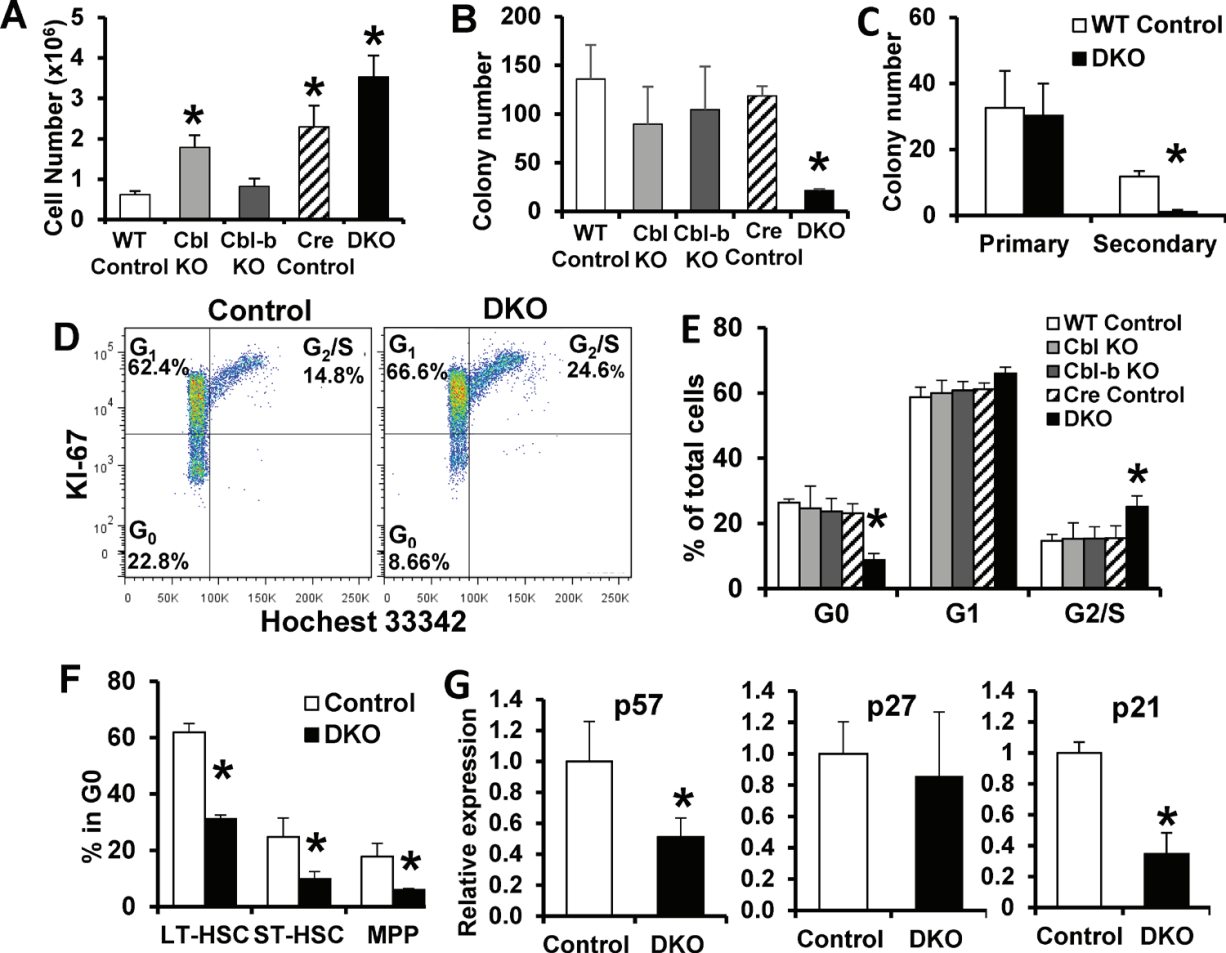
Increased cycling and reduced quiescent HSC fractions in Cbl/Cbl-b DKO mice **(A–B)** LTC-IC assay – cell counts after initial co-culture (A), and subsequent colony-forming assays (B) Results from three experiments are pooled; 2 replicates in each experiment. **(C)** Serial colony-forming assay. Results from three experiments are pooled; 3 replicates in each experiment. **(D–F)** Cell cycle analysis. Representative FACS plots are shown in (D) Quantification of cell cycle phases for total LSKs (E); LT-HSC, ST-HSC and MPP subpopulations (F) Data from at least three experiments are pooled and shown as mean ± SD. **(G)** qRT-PCR of p57, p27 and p21 mRNA in LT-HSCs normalized using GAPDH. Data from four independent repeats are pooled and shown as mean ± SD (*p < 0.05).

Cbl-KO HSCs were reported to have an improved reconstitution ability [22]. As only the Cbl/Cbl-b DKO mice succumb to lethal MPD [21] (and above results), we examined the impact of Cbl/Cbl-b DKO on HSC maintenance. In long term culture-initiating cell assays (LTC-IC), DKO bone marrow mononuclear cells (BMMNCs) co-cultured with OP-9 stromal cells for 2 weeks predictably proliferated to a greater extent compared to cells of other genotypes (Figure 2A); when sorted and re-plated, the DKO BMMNCs showed a significantly lower colony-forming ability vs. controls or individual KO cells (Figure 2B). In a serial re-plating assay, sorted control and DKO LSK cells showed comparable colony-forming ability during initial plating, but DKO LSK cells showed significantly lower colony formation upon re-plating (Figure 2C). Minimal differences between control and DKO LSK proliferation during initial plating reflects the inclusion of 5 cytokines in media (Figure S3A).

In view of these *in vitro* results, we analyzed the cell cycle status of HSCs freshly isolated from control and Cbl/Cbl-b DKO mice using FACS (Figure 2D and 2E). While WT control, Cbl-KO, Cbl-b-KO and Cre control LSKs showed comparable cell cycle profiles, DKO LSKs showed a significant decrease in the G_0_ (Ki-67^−^, Hochest33342^low^) fraction, with an increase in the G_1_ (Ki-67^+^, Hochest33342^low^) and G_2_/S (Ki-67^+^, Hochest33342^high^) fractions. Further analysis showed that the G_0_ fraction was significantly reduced in all major subsets of DKO LSKs, including the long-term HSCs (LT-HSC ; CD34^−^, FLT3^−^, LSK), short-term HSCs (ST-HSC; CD34^+^, FLT3^−^, LSK) and multipotent progenitors (MPP; CD34^+^, FLT3^+^, LSK) (Figure 2F). Use of alternate LT-HSC markers (CD48^−^, CD150^+^, LSK) confirmed these findings (Figure S3B).

Cip/Kip family of CDK inhibitors, including p57, p27 and p21, are critical cell cycle regulators that enforce HSC quiescence [32–34]. At the mRNA level, p57 is more enriched in LT-HSCs compared to ST-HSCs, MPPs or committed progenitors, while p27 expression does not differ [34]. Our real-time qPCR revealed significantly reduced p57 and p21 expression in DKO LT-HSCs compared to WT LT-HSC, which suggests loss of quiescence in DKO LT-HSC (Figure 2G). Altogether, our results demonstrate that Cbl/Cbl-b DKO HSCs are defective in the maintenance of quiescence under conditions of homeostasis.

### Deregulation of c-Kit and FLT3 signaling in DKO HSCs

**Figure 3:**
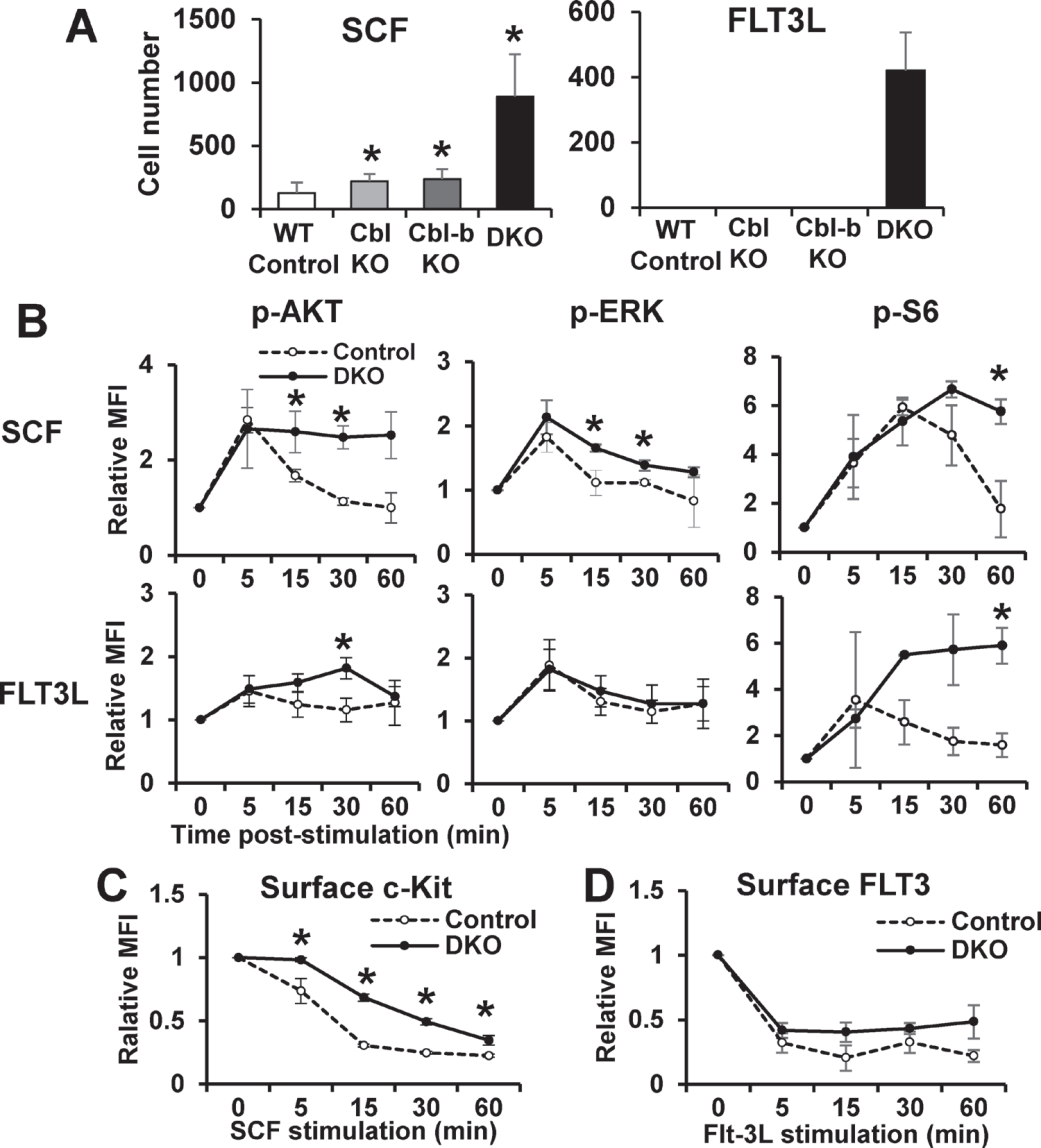
Loss of Cbl and Cbl-b enhances c-Kit and FLT3 signaling in HSCs **(A)** Proliferation - LSK cells (50/well of 96-well plate; 6 replicates in each experiment) were cultured with SCF or FLT3L (10 ng/ml) for 7 days and counted. Representative experiment of 2. **(B)** p-Akt, p-Erk and p-S6 in LSK cells after time-course ligand stimulation were analyzed by FACS. Data expressed as mean fluorescence intensity (MFI) plots (mean ± SD of at least two independent experiments). **(C–D)** Cell surface c-Kit (C) and FLT3 (D) levels in LSKs were quantified by FACS at various time points after SCF or FLT3L stimulation. Data from at least 2 independent experiments (*p < 0.05).

c-Kit and FLT3 play essential roles in HSC quiescence [3, 35]. Previous studies in model cell lines have shown these RTKs to be targets of Cbl [12], but whether they are targets of Cbl proteins in HSCs is unknown. We therefore assessed if Cbl and Cbl-b proteins negatively regulate c-Kit and FLT3 signaling in HSCs. Indeed, Cbl/Cbl-b DKO LSKs hyper-proliferate in the presence of SCF or FLT3L, compared to control LSKs, while single KO LSKs exhibit significantly less hyper-proliferation (Figure 3A). Using FACS analysis with surface markers and phospho-specific antibodies, we quantified Akt, Erk and S6 kinase phosphorylation in the LSK compartment following *in vitro* SCF or FLT3L stimulation. While initial ligand-induced phosphorylation signals were comparable between WT and DKO LSKs, the signals were significantly more sustained (15 min and beyond) in DKO LSKs (Figure S4), a trend seen across multiple experiments (Figure 3B). Since Cbl proteins mediate ligand-induced RTK downregulation from the cell surface [12], we compared the cell surface c-Kit and FLT3 levels using FACS on WT vs. DKO LSK cells at various times after ligand stimulation. Slower downregulation of both c-Kit and FLT3 was observed across multiple experiments in DKO LSKs albeit only the c-Kit downregulation differences were statistically significant (Figure 3C and 3D). Together, these findings establish that Cbl/Cbl-b proteins function as essential negative regulators of c-Kit and FLT3 signaling in HSCs.

### Modulating c-Kit and FLT3 signaling impacts DKO HSC self-renewal ability

**Figure 4:**
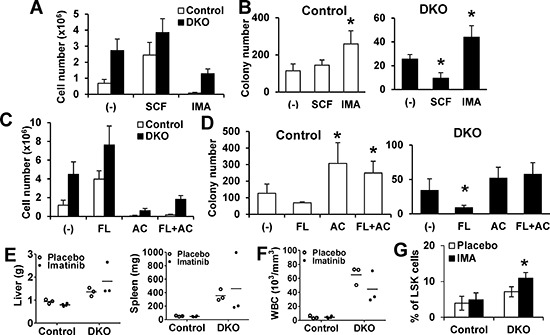
Impact of modulating c-Kit or FLT3 signaling on DKO HSC exhaustion **(A–B)** LTC-IC assays. Initial co-cultures performed without growth factors (control), or with SCF (100 ng/ml) or Imatinib (1 uM), and cell numbers counted (A), followed by colony forming assay (B) Data are from four independent experiments. **(C–D)** Assay was performed as in A and B, with primary co-culture without (Control), or with FLT3L (FL, 100 ng/ml), AC220 (AC, 100 nM) or FLT3L (100 ng/ml) + AC220 (100 nM). Cell numbers after initial culture (C) and colony-formation assay (D) are shown. Data are from four independent experiments. **(E–G)** Imatinib *in vivo* treatment. (E) Liver and spleen weights of mice with indicated treatment. (F) WBC counts. Dots represent individual mice. (G) BM analysis.% LSK cells in Lin- cells is shown. Data from at least three mice are pooled and shown as mean ± SD. *p < 0.05.

To link the Cbl/Cbl-b mediated negative regulation of c-Kit and FLT3 to their role in maintaining HSC self-renewal, we assessed the impact of growing BMMNC in the presence of activating ligands or kinase inhibitors of these RTKs on subsequent colony-forming ability in LTC-IC assays. Both control and DKO cells proliferated in SCF while c-Kit inhibitor imatinib abrogated (WT) or reduced (DKO) proliferation (Figure 4A). The subsequent colony-forming ability of unstimulated or SCF-expanded WT cells was comparable, while imatinib-cultured cells showed slightly higher colony-forming ability (Figure 4B). In contrast, SCF-expanded DKO cells showed significantly reduced colony-forming ability vs. the unstimulated DKO cells, and imatinib pre-treatment restored it (Figure 4B). Similar results were seen with growth in FLT3L +/− FLT3 inhibitor AC220 (Figure 4C and 4D), with the minor difference that FLT3-treated control cells showed a slightly reduced colony-forming ability.

As c-Kit or FLT3 inhibition markedly inhibited DKO BMMNC proliferation, yet preserved their colony forming ability *in vitro*, we assessed the effect of imatinib treatment on MPD in Cbl/Cbl-b DKO mice (Figure S5A). However, a 3-week course of imatinib failed to ameliorate the MPD in DKO mice, including hepatosplenomegaly and PB counts (Figure 4E and 4F, Figure S5B and S5C). Interestingly, analysis of BM in imatinib-treated DKO mice showed a dramatic increase in the LSK compartment (Figure 4G and Figure S5D), and increased colony forming ability *in vitro* (Figure 4A and 4B). These features are consistent with lack of an impact of imatinib treatment. Collectively, our data indicate that negative regulation provided by Cbl and Cbl-b is required for c-Kit and FLT3 dependent HSCs maintenance.

### DKO HSCs show impaired reconstitution ability *in vivo*

**Figure 5:**
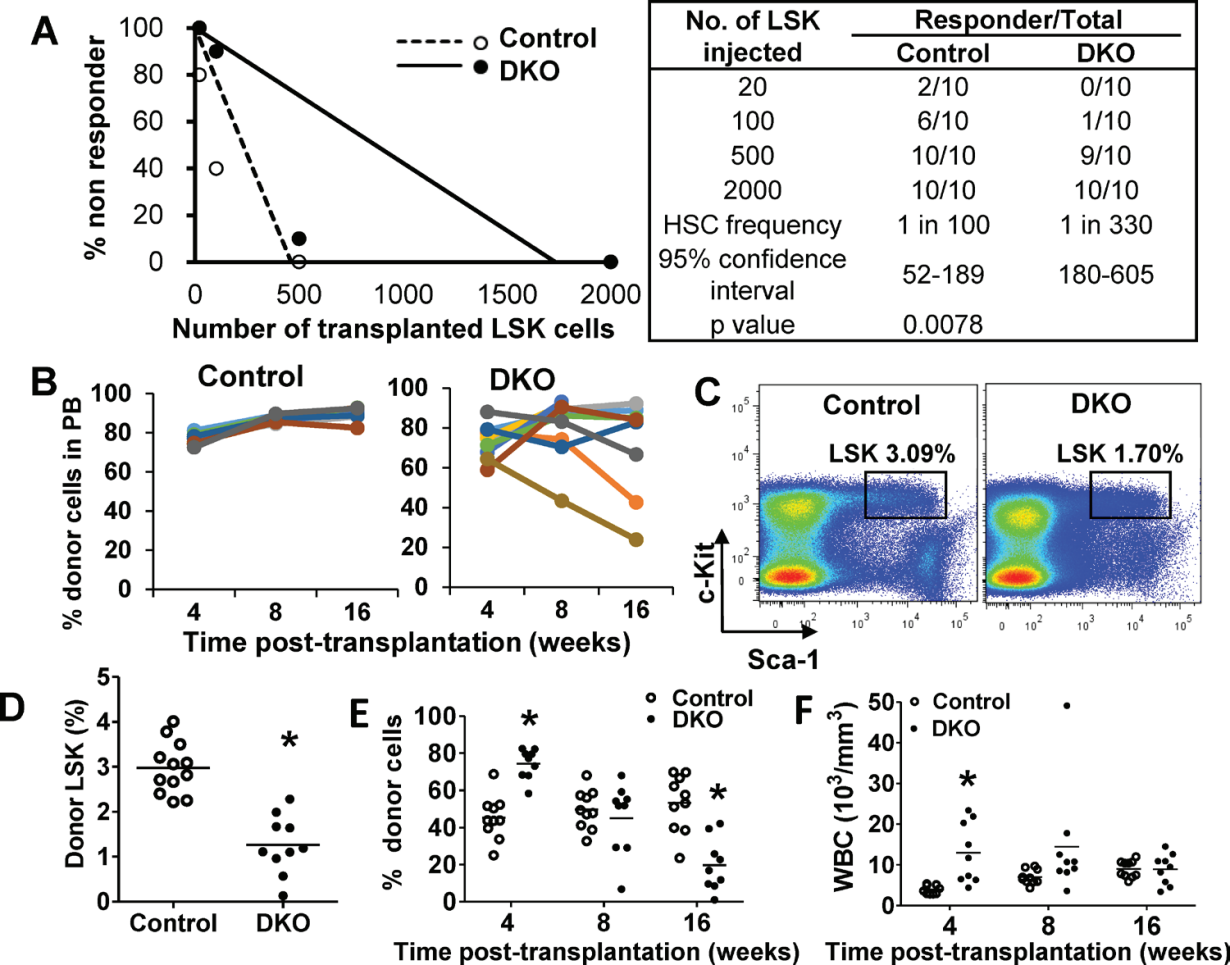
Cbl/Cbl-b DKO HSCs show impaired *in vivo* reconstitution ability **(A)** HSC limiting-dilution transplantation. Indicated numbers of LSKs were transplanted and mice with < 0.5% donor-derived chimerism at 16 weeks were considered non-responders. Data from two experiments are pooled. **(B)** 2000 LSKs were transplanted and donor-cell chimerism in PB analyzed at the indicated times. Data from two experiments are pooled. **(C–D)** Donor LSK pool was analyzed in recipient BM at around 20 weeks. Representative FACS plot (C) and quantitation (D) are shown only for recipients with > 70% donor cell reconstitution. Data from three experiments are pooled. **(E–F)** Secondary transplants with 2000 donor LSKs sorted from primary recipients transplanted 20 weeks earlier. Donor cell chimerism in PB **(G)** and WBC counts **(H)** over time are shown. Data from two repeats are pooled (*p < 0.05).

Given our results that DKO HSCs exhibit impaired colony forming ability *in vitro*, we assessed the self-renewal ability of DKO vs. WT HSCs *in vivo*. We transplanted limiting dilutions of freshly-sorted WT or DKO LSKs, assessed engraftment at 16 weeks using peripheral blood analyses, and calculated the HSC frequency. Compared to a frequency of 1:100 for WT control, the HSC frequency of DKO LSK cells was significantly reduced (1:300) (Figure 5A). Next, we transplanted 2000 WT or DKO LSKs (sufficient for engraftment) and assessed the proportion of donor cells in PB at 4, 8 and 16 weeks post-transplant (Figure 5B). Compared to expected sustained reconstitution with WT LSKs, DKO LSK recipients showed a more variable albeit substantial reconstitution at 4 weeks; notably, several DKO LSK recipients exhibited a decrease in donor-derived WBCs with time, indicating impaired ability of DKO LSK cells to maintain long-term reconstitution. We further analyzed DKO LSK recipients that showed high donor cell reconstitution (> 70%) at 16 weeks post-transplant (to exclude mice that may have received a transplant with predominantly ST-HSC and MPPs, populations with only a short-term reconstitution ability) (Figure S6A). Notably, these mice exhibited a significantly smaller BM LSK compartment compared to WT LSK recipients (Figure 5C and 5D). Thus, even in recipients with robust reconstitution, DKO HSCs were unable to maintain the HSC pool.

Since the absolute numbers of donor LSK cells were comparable in primary WT and DKO LSK transplant recipients (Figure S6B), we used secondary HSC transplantation to further compare the WT vs. DKO HSC reconstitution ability. 2000 LSKs from mice transplanted 20 weeks earlier and showing 70% or higher donor cell chimerism in PB, were transplanted together with helper cells into secondary recipients. DKO LSK recipients again showed robust reconstitution at 4 weeks; however, the DKO donor-derived progeny had declined by 8 weeks and became significantly lower by 16 weeks (Figure 5E), suggesting the exhaustion feature of DKO donor cells in secondary transplant. In contrast, control LSK recipients maintained the level of donor cells. Furthermore, leukocytosis in secondary DKO LSK recipients was obvious compared to WT LSK recipients at 4 weeks but diminished by 8 weeks in a majority of mice, and disappeared by 16 weeks (Figure 5F).

Altogether, these results further support the conclusion that Cbl/Cbl-b are required for the maintenance of HSCs by promoting quiescence, and abrogation of this mechanism promotes HSC exhaustion.

### 5-Fluorouracil (5-FU) accelerates loss of DKO HSC progeny and ameliorates MPD

**Figure 6:**
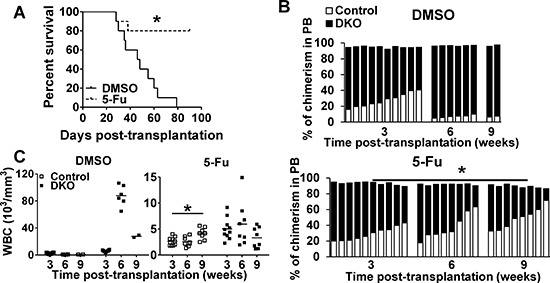
5-FU promotes DKO HSC exhaustion **(A)** Survival curve. **(B)** Donor cell chimerism in recipients treated with DMSO (upper) or 5-FU (lower) at the indicated time points post-transplantation. Each bar represent one individual mice. **(C)** Peripheral WBC counts in recipients corresponding to those derived from control or DKO donor cells after treatment with DMSO control (left) or 125 mg/kg 5-FU (right) treatment at the indicated times post-transplantation. Data pooled from two experiments. *n* = 10, *p < 0.05.

Findings above indicated that Cbl/Cbl-b DKO impairs HSC quiescence and self-renewal while expanding the proliferative stem and progenitor compartments. The anti-leukemic drug 5-FU selectively eliminates cycling HSCs and progenitors [36]. Therefore, we reasoned that 5-FU treatment of Cbl/Cbl-b DKO mice will accelerate DKO HSC exhaustion and ameliorate MPD. WT Control and DKO BM cells were co-transplanted into lethally-irradiated recipients at a 1:1 ratio followed by treatment of recipients with DMSO or 5-FU at 3 and 6 weeks post-transplantation (Figure S7). DMSO-treated mice developed a rapidly-lethal MPD (Figure 6A). In contrast, 5-FU treatment significantly improved the overall survival of DKO transplant recipients (Figure 6A), and led to a steady decrease in the proportion of DKO WBCs in PB (Figure 6B). Furthermore, while untreated DKO recipients showed a steady increase in the proportion of peripheral blood WBCs of DKO origin relative to those of WT origin, a reverse trend was observed in 5-FU treated mice (Figure 6C). These results further support our conclusion that Cbl and Cbl-b are essential to maintain HSC quiescence and self-renewal.

## DISCUSSION

PTK-coupled HSC receptors activated by niche-derived growth factors are required to maintain HSC quiescence and self-renewal [3, 6, 35, 37]. Yet mutational activation of these receptors or their signaling components is linked to leukemic disorders [7–10]. Regulatory mechanisms that dictate such distinct outcomes of PTK-associated receptor signaling are unclear. Using a genetic approach, we establish that PTK-directed ubiquitin ligases Cbl and Cbl-b are essential for HSC quiescence and maintenance. We also show that Cbl and Cbl-b provide a key switch to determine HSC self-renewal versus proliferative exhaustion outcomes of the activation of c-Kit and FLT3, RTKs essential for HSC quiescence and self-renewal. Notably, the ability of clinically used anti-leukemic drug 5-FU to accelerate exhaustion of Cbl/Cbl-b DKO HSCs and to significantly ameliorate the associated lethal MPD outcome raises the potential of a novel “exhaustion” based therapy of certain malignancies such as those associated with Cbl mutations.

We demonstrate that all HSC subsets express Cbl and Cbl-b, and that MMTV-Cre induces Cbl deletion in all subsets (Figure S1E). Notably, only Cbl/Cbl-b DKO mice exhibit major functional perturbations of HSCs, with a lethal MPD that can be transferred via BM transplant, even though Cbl-KO (shown here and [22]), Cbl-b-KO, or Cbl-KO/Cbl-b^+/−^ (Cre control) mice show HSC expansion. Furthermore, only the HSCs, and not the committed progenitors of DKO mice can transfer the MPD. Thus, Cbl and Cbl-b play a redundant but essential role as cell-autonomous regulators of HSCs.

Notably, one feature of human MDS/MPN patients is anemia, which is recapitulated in Cbl/Cbl-b DKO mice (Figure S1A) but not in transplanted mice (Figure 1A). Our current thinking is that anemia is a manifestation of the late stage of MPD. Cbl/Cbl-b DKO mice (Figure S1A) have had MPD for several weeks prior to analyses, which leads to more obvious anemia. However, in transplanted mice (Figure 1A), donor BM cells re-establish the hematopoietic system around 3 weeks after transplantation [38] and blood cells counts was obtained at 4 week after transplantation. One week of MPD might only interfere with erythropoiesis to a limited extent. Notably, even in the transplant model (Figure 1A), albeit not significant, DKO mice exhibit a slight decrease of RBC compared to other controls.

By analyzing the cell cycle distribution in bone marrow cell compartments, we identified a marked increase in cycling (G1 and G2/M) LT-HSC, ST-HSC and MMPs, and a marked reduction in their quiescent (G_0_) fractions (Figure 2F) in DKO mice, thus revealing a novel role of Cbl proteins to help maintain HSCs quiescence under homeostasis. Aside from pathways that restrict the cell cycle traverse, such as the Rb-family and CDK inhibitor proteins [39], it is now clear that active signals received from the microenvironment are required to maintain HSC quiescence [1]. Relevant to the role of Cbl proteins, activation of PTK-coupled receptors such as Tie-2, c-Mpl, c-Kit and FLT3 [3–6] is involved in HSC quiescence. Proximally-acting regulators of RTK signaling in HSCs that dictate a quiescence outcome are unknown. Impaired ligand-induced c-Kit and FLT3 downregulation (Figure 3C and 3D) and sustained downstream signaling (Figure 3B) in DKO HSCs provide the first evidence that Cbl proteins negatively regulate RTKs in normal HSCs. Notably, ligand-induced downregulation of FLT3 in DKO HSC is less obvious compared to that of c-Kit (Figure 3D). We speculate that FLT3 may be regulated in its trafficking mechanism differently compared to c-KIT. For example, other ubiquitin ligases may play some role or Cbl proteins may function by primarily regulating downstream signaling components which are known to interact with Cbl proteins and be targeted for downregulation. The latter idea is consistent with significant impact of Cbl/Cbl-b deficiency on pAKT and pS6 levels over time (Figure 3B).

Consistent with the conclusion that Cbl proteins negatively regulate RTKs in HSCs, Cbl and Cbl-b DKO HSCs hyper-proliferate in response to SCF or FLT3L (Figure 3A). Amelioration of MPD in Cbl RF-KI mice by FLT3L gene deletion [24] is consistent with our findings. Since Cbl proteins only target RTKs once these have been activated [12], we suggest that Cbl and Cbl-b serve to physiologically limit the duration of signaling to a level sufficient for survival of HSCs but suboptimal for their cell cycle entry. Hyperactive PI3K pathway signaling, as seen with sustained p-AKT and p-S6 upon ligand activation (Figure 3B), is one likely mediator of reduced quiescence in DKO HSCs since PTEN, a direct PI3K pathway attenuator, is required for efficient HSC quiescence and self-renewal [40].

Loss of quiescence-promoting factors is known to promote in HSC exhaustion, reflecting a tight linkage between HSC quiescence and self-renewal [32–34]. Using both *in vitro* and *in vivo* models, we show that loss of Cbl and Cbl-b promotes HSC exhaustion. *In vitro*, colony-forming ability of BM cultures in SCF or FLT3L was severely diminished in DKO HSCs (Figure 4A–4D). The impaired self-renewal of DKO HSCs was a direct consequence of exaggerated receptor signaling, as demonstrated by the restoration of self-renewal with c-KIT or FLT3 specific kinase inhibitors (Figure 4B, 4D). Transplant studies demonstrated that Cbl/Cbl-b DKO HSCs, which were competent for engraftment, were impaired in maintaining hematopoiesis over time and during serial transplantation (Figure 5E, 5F). Interestingly, HSCs from Cbl-KO mice were shown to have increased self-renewal ability [22]. Notably, Cbl-KO HSCs are distinctly less hyper-proliferative to SFC or FLT3L (Figure 3A), suggesting that Cbl-b expression in Cbl-KO mice is sufficient to maintain HSC self-renewal. However, since Cbl and Cbl-b have some unique biochemical interactions [12], the possibility that Cbl-b preferentially regulates HSC quiescence and self-renewal needs to be addressed in future studies.

Even though all HSC subpopulations expand and show reduced quiescence in Cbl/Cbl-b DKO mouse BM, they show skewed progenitor expansion with myeloid and lymphoid cell expansion in the periphery while platelets and RBCs are not increased (also seen upon disease transfer). The mechanisms of skewing remain unclear. The hyper-responsiveness of DKO (Figure 3A) or Cbl-RF-KI [24] HSCs to FLT3L may promote such skewing, since Flt3L was shown to instruct HSCs toward myeloid-lymphoid lineage and not the megakaryocyte-erythrocyte lineage [4]. Loss of Cbl was reported to facilitate the transition of more primitive, c-Kit-low HSCs into less primitive c-Kit-high HSCs [3]. This mechanism is consistent with the restoration of *in vitro* colony forming ability of DKO HSCs by imatinib or AC220 treatment (Figure 4A–4D) and the poor outcome of imatinib treatment in DKO mice (Figure 4E–4G). While we did not see an increase in surface c-Kit expression in DKO HSCs, the possible role of elevated c-Kit signaling in DKO HSCs as a mechanism to promote HSC transition to a less primitive and possibly less quiescent state needs further study.

5-FU is a clinically used anti-leukemic DNA base analog that is selectively cytotoxic to cycling cells [36]. Since more HSCs in DKO mice are actively cycling, at the expense of the quiescent HSC pool, we reasoned that elimination of these cycling HSCs with 5-FU will ameliorate the MPD induced by DKO HSCs. Indeed, 5-FU treatment suppressed the MPD phenotype of DKO bone marrow transplant recipients, with progressive loss of the DKO donor cell progeny in PB, and significantly increased their survival (Figure 6A). These results are in contrast to the impact of imatinib treatment, which failed to ameliorate the MPD in DKO mice, and in fact led to an expansion of the HSC (LSK) compartment (Figure 4E–4G). This result is consistent with our *in vitro* results showing restoration of self-renewal ability of DKO HSCs *in vitro* (Figure 2A and 2B). Recently, multi-kinase inhibitor Dasatinib was reported to induce transient expansion of quiescent HSCs in WT mice as well as in Cbl RING finger knock in model of MPD [41]. These studies are consistent with our observations, and the model that Cbl proteins regulate HSC quiescence, although direct comparisons of the different models are needed to establish this firmly.

Although gene deletions are absent in patients, mutations in leukemic patients are commonly duplicated and these are thought to effectively compete with the endogenous Cbl-b (and remaining WT Cbl in rare heterozygous patients), consistent with the failure of RF-KI mouse model to develop MPD unless the WT Cbl allele is deleted [24]. Thus, a hematopoietic stem cell knockout of Cbl and Cbl-b functionally mimics this state, providing a model to study mutant Cbl-associated leukemogenesis. Indeed, the leukemic disease in Cbl/Cbl-b DKO mice resembles that in human MPN/MDS and recapitulates the consequence hypothesized for the dominant negative inhibition of Cbl mutations [14, 21]. It will be of interest to investigate whether or not the leukemias with mutant Cbl are associated with reduced HSC self-renewal since some juvenile myelomonocytic leukemia patients show spontaneous resolution [15].

Notably, HSC exhaustion due to hyperproliferation has been shown in BCR-Abl driven leukemia [42]. Our findings that 5-FU targeting of actively cycling HSCs in DKO mice ameliorates the MPD and prolongs survival is of considerable future interest. Should human leukemia patients with Cbl mutations exhibit impaired HSC quiescence and self-renewal, as shown with Cbl/Cbl-b DKO mice here, targeting of their actively cycling leukemogenic HSCs could provide a novel “exhaustion” therapy for these patients.

## MATERIALS AND METHODS

### Animals

Cbl-KO [31], Cbl-b-KO [25] and MMTV-Cre-based Cbl/Cbl-b-DKO (DKO) [21] strains were maintained on a C57/Bl6 background and genotyped using tail DNA PCR with specific primers (Supplementary Table 2). WT and Cre control (Cbl^flox/flox^, Cbl-b^+/−^; MMTV-Cre) mice were littermates identified during breeding. B6.SJL-Ptprca Pepcb/BoyJ mice (The Jackson Laboratory) were used as transplant recipients. Mice were housed under specific pathogen-free conditions and sacrificed for analysis around 8 weeks of age, unless otherwise specified. Mouse studies were approved by the UNMC IACUC.

### Bone marrow preparation and FACS analysis

Bone marrow cells were harvested from femurs and tibiae. Lin^−^ cells were MACS-purified (Miltenyi) and stained with fluorescent antibodies for cell analysis or sorting. For cell cycle and phospho-Flow analyses by flow cytometry, Lin^−^ cells were fixed, permeablized and stained with the indicated antibodies, and analyzed on a BD LSRII or Aria II. Data were analyzed using FlowJo software (Tree Star). Detailed procedures and reagents are included in supplementary information.

### RNA isolation and quantitative real-time PCR analysis

RNA extracted from FACS-sorted cells (RNAqueous-Micro Kit, Life Technologies) was reverse-transcribed (QuantiTect Reverse Transcription Kit, Qiagen) and subjected to quantitative real-time PCR (QuantiTect SYBR Green Kit, Qiagen) on a BioRad CFX96 thermocycler, following the manufacturer's instructions. Primers are listed in Supplementary Table 3.

### Histopathology

Organs were formalin-fixed, dehydrated in 70% EtOH, paraffin-embedded and H&E stained. Whole blood CBCs were performed on a Scil Vet abc Animal Blood Counter (Scil Animal Care).

### Colony-forming assays

For long-term culture-initiating cell (LTC-IC) assay, BM cells were co-cultured with pre-irradiated OP-9 stromal cells (ATCC) followed by a colony-forming assay. For the serial-plating assay, FACS-sorted LSK cells were cultured in MethoCult M3234 (Stemcell Tech.) with 5-cytokines (SCF, TPO, IL-3, IL-6 and FLT3-L; from Peprotech) for 7 days, followed by colony counting. Cells were collected and plated for secondary colony-forming assays. See supplementary information for details.

### Bone marrow transplantation assays

8-week old B6.SJL-Ptprca Pepcb/BoyJ mice (CD45.1) were lethally-irradiated and transplanted 24 h later with donor cells from age/gender-matched mice (CD45.2) together with helper BM cells (CD45.1 and CD45.2 heterozygous). See supplementary information for details.

### Statistics

Unpaired Student's *t* test on Prism was used to calculate the *p* values.

## CONCLUSION

Our studies, using inducible Cbl-KO on a Cbl-b-null background to induce Cbl/Cbl-b DKO in HSCs, reveal a previously unknown yet physiologically essential role of Cbl and Cbl-b in the maintenance of HSC quiescence and self-renewal. Our studies provide a mechanistic explanation for how certain growth factors, working through PTK-coupled cell surface receptors, can maintain HSC quiescence, while hyperactive signaling downstream of the same receptors promotes hematopoietic malignancies. We suggest that activation-dependent recruitment of Cbl and Cbl-b serves to curtail PTK-coupled receptor signaling to restrain HSCs from committing into proliferation while allowing some functions such as cell survival. Such a balance would favor HSC self-renewal, and its abrogation can promote HSC exhaustion and leukemic states. While we have focused on c-Kit and FLT3 signaling, Cbl proteins may also regulate other PTK-coupled receptors, such as Tie-2 and c-Mpl [1], to fully coordinate HSC quiescence maintenance by niche-derived growth factors.

## SUPPLEMENTAL DATA


