# Supplementary material for: Loss of Cbl and Cbl-b ubiquitin ligases abrogates hematopoietic stem cell quiescence and sensitizes leukemic disease to chemotherapy
Source: Oncotarget. 2015 Mar 19;6(12):10498–509. doi: 10.18632/oncotarget.3403 (PMC4496370; doi:10.18632/oncotarget.3403)
Supplement: Supplementary file 1 [file oncotarget-06-10498-s001.pdf]

SUPPLEMENTAL DATA

SUPPLEMENTARY EXPERIMENTAL PROCEDURES

Reagents and antibodies

The murine recombinant cytokines SCF, TPO, IL-3, IL-6 and FLT3L were obtained from PeproTech. MethoCult M3234 Methylcellulose medium was obtained from Stem Cell Technologies. The following antibodies for flow cytometry were obtained from eBioscience: CD45.1 (A20); CD45.2 (104); Sca-1 (D7); c-Kit (2B8); CD16 (93), CD48(HM48-1), Ki-67(SolA15) and ILR7(A7R34). CD34 (RAM34) and FLT3 (A2F10.1) antibodies were obtained from BD Biosciences. CD150 (TC15-12F12.2) antibody was obtained from Biolegend. The following antibodies were obtained from Cell Signaling Technology: p-Erk 1/2 (D13.14.4E), p-Akt (D9E) and p-S6 (D57.2.2E). Fetal bovine serum (FBS) was from Hyclone. Imatinib and AC220 were from LC Laboratory. Biochemicals were from Sigma or Life Technologies unless indicated. OP-9 stromal cell line was obtained from ATCC and maintained by culture in αMEM (Gibco) with 10% FBS.

BM preparation and FACS analysis

Whole bone marrow cell suspensions were prepared from femurs and tibiae. For stem and progenitor cell analysis and sorting, mature hematopoietic cells (lineage-positive cells) were labeled with antibodies against CD5, B220, CD11b, Gr-1, and 7-4 (mouse lineage depletion kit; Miltenyi Biotechnology) and magnetically depleted using the autoMACS (Miltenyi Biotechnology). Lineage-negative cells were then stained with antibodies followed by cell analysis or sorting. Flow cytometry was performed on a BD LSRII or Aria II at the UNMC Flow Cytometry Research Facility. Data were analyzed using FlowJo software (Tree Star). Cell populations were defined as follows[1]: LT-HSC: $CD34^-$ $FLT3^-$ Lin^- $Sca\text{-}1^+$ $c\text{-}Kit^+$; ST-HSC: $CD34^+$ $FLT3^-$ Lin^- $Sca\text{-}1^+$ $c\text{-}Kit^+$; MPP: $CD34^+$ $FLT3^+$ Lin^- $Sca\text{-}1^+$ $c\text{-}Kit^+$; LSK: Lin^- $Sca\text{-}1^+$ $c\text{-}Kit^+$; CMP: $CD16^-$ $CD34^+$ Lin^- $Sca\text{-}1^-$ $c\text{-}Kit^+$; GMP: $CD16^+$ $CD34^+$ Lin^- $Sca\text{-}1^-$ $c\text{-}Kit^+$; MEP: $CD16^-$ $CD34^-$ Lin^- $Sca\text{-}1^-$ $c\text{-}Kit^+$; CLP: $IL\text{-}7R^+$ $FLT3^+$ Lin^- $Sca\text{-}1^{low}$ $c\text{-}Kit^{low}$.

Cell cycle analysis

Bone marrow-derived lineage-negative cells were isolated using autoMACS and stained with the cell surface markers Sca-1 and c-Kit. After staining, cells were fixed in 2% paraformaldehyde, washed and permeabilized in PBS containing 0.5% saponin, 0.25% Triton X-100, 2% FBS, and 2% BSA for intracellular staining. Cells were then stained with Hoechst 33342 at 40ug/ml (Sigma) and Ki-67 antibody. After washing, cells were analyzed on BD LSRII. Data were analyzed using FlowJo software (Tree Star).

FACS-based phosphorylation (phospho-Flow) analysis

For phospho-Flow analyses, lineage-negative cells were stained with anti-Sca-1 antibody and incubated for one hour in serum-free IMDM medium at 37°C before stimulation with mouse SCF or FLT3 ligand. At the indicated times, cells were fixed and permeabilized, as described in methods followed by staining for c-Kit together with anti-phospho-Erk and anti-phospho-Akt antibodies. Cells were washed and analyzed on BD LSRII. To assess surface c-kit or FLT3 levels after SCF or FLT3L stimulation respectively, lineage-negative cells were incubated in growth factor-free IMDM at 37°C for 1 hour and stained with anti-Sca-1 antibody followed by ligand stimulation for the indicated times. Cells were then fixed with 2% paraformaldehyde, washed and labeled with antibodies against surface c-Kit or FLT3 before analyses. Data are shown as the mean fluorescence intensity expressed as a % of unstimulated control.

In vitro assays

For the long-term culture-initiating cell (LTC-IC) assay, 2×10^5 bone marrow-derived mononuclear cells were co-cultured with pre-irradiated OP-9 stromal cells in IMDM medium (Gibco) supplemented with 12.5% fetal bovine serum (FBS) (Hyclone), 12.5% horse serum (Lonza) and 10^{-8} M dexamethasone (Sigma). After 2 weeks, total hematopoietic cells were counted with a hemocytometer and the $CD45.2^+$ cells were FACS-sorted. In the subsequent colony-forming assays, 3×10^4 sorted cells were mixed with 1ml of MethoCult M3234 medium supplemented with cytokines (SCF 50 ng/ml, TPO 20 ng/ml, IL-3 10 ng/ml, IL-6 10 ng/ml and FLT3-L 10 ng/ml, referred as 5-cytokines combination) and plated in 35 mm tissue culture plates (Falcon). Colonies that have more than 50 cells were visually counted on day 7. For the serial plating assay, FACS-sorted LSK cells were used for colony-forming assays. 50 LSK cells per 35 mm plate were cultured in 1 ml of MethoCult M3234 medium with 5-factor combination for 7 days. Colonies were counted as above, cells were collected and 1×10^4 cells were plated for a second round of colony forming assays.

Bone marrow transplantation assays

For all transplantation experiments, 8-week old B6.SJL-Ptprca Pepcb/BoyJ mice (CD45.1) were used as

recipients. Recipients were lethally-irradiated (1100 rad in two split doses with 3 hours interval) 24 hours before transplantation. Donor cells were derived from 8-week old gender-matched mice (CD45.2) of the genotypes indicated in various experiments. For whole BM transplantation, 2×10^6 donor BM cells were transplanted. For purified BM population transplants, the indicated numbers of LSK, LKS^- and Lin^+ $c\text{-}kit^-$ cells isolated by FACS-sorting were transplanted together with 2×10^5 helper BM cells (heterozygous at CD45.1 and CD45.2, radio-protection). For HSC serial diluting transplantation (non-responder assays) to assess functional HSC frequency, the indicated numbers of FACS-sorted LSK cells were transplanted together with 2×10^5 helper cells. The chimerism of donor cells in peripheral blood was calculated using L-Calc™ Software (STEMCELL Technologies) to assess the percentage of non-responder mice. For serial transplantation, 2000 donor-derived LSK cells were sorted from primary recipients at around 20 weeks after primary transplantation and transplanted into secondary recipients. Peripheral blood was obtained by submandibular vein bleeding at 4, 8 and/or 16 weeks post-transplantation and engraftment efficiency in recipients was monitored based on the chimerism of $CD45.2^+$ cells in peripheral blood. 0.5% or higher donor-derived cells in the peripheral blood were scored as responders.

Imatinib and 5-Fluorouracil *in vivo* treatment

For Imatinib treatment, mice were treated with placebo (PBS) or Imatinib in a volume of 100 ul PBS (100 mg/kg). Treatment was administered by oral gavage injection twice daily until the end of the experiment. For 5-Fu treatment, 5-Fu was prepared as 50 mg/ml solution in DMSO and diluted in PBS. Treatment was administered by i.p. injection at indicated time at the dose of 125 mg/kg.

SUPPLEMENTARY REFERENCES

1. Seita J, Weissman IL. Hematopoietic stem cell: self-renewal versus differentiation. Wiley Interdiscip Rev Syst Biol Med. 2010; 2:640–653.

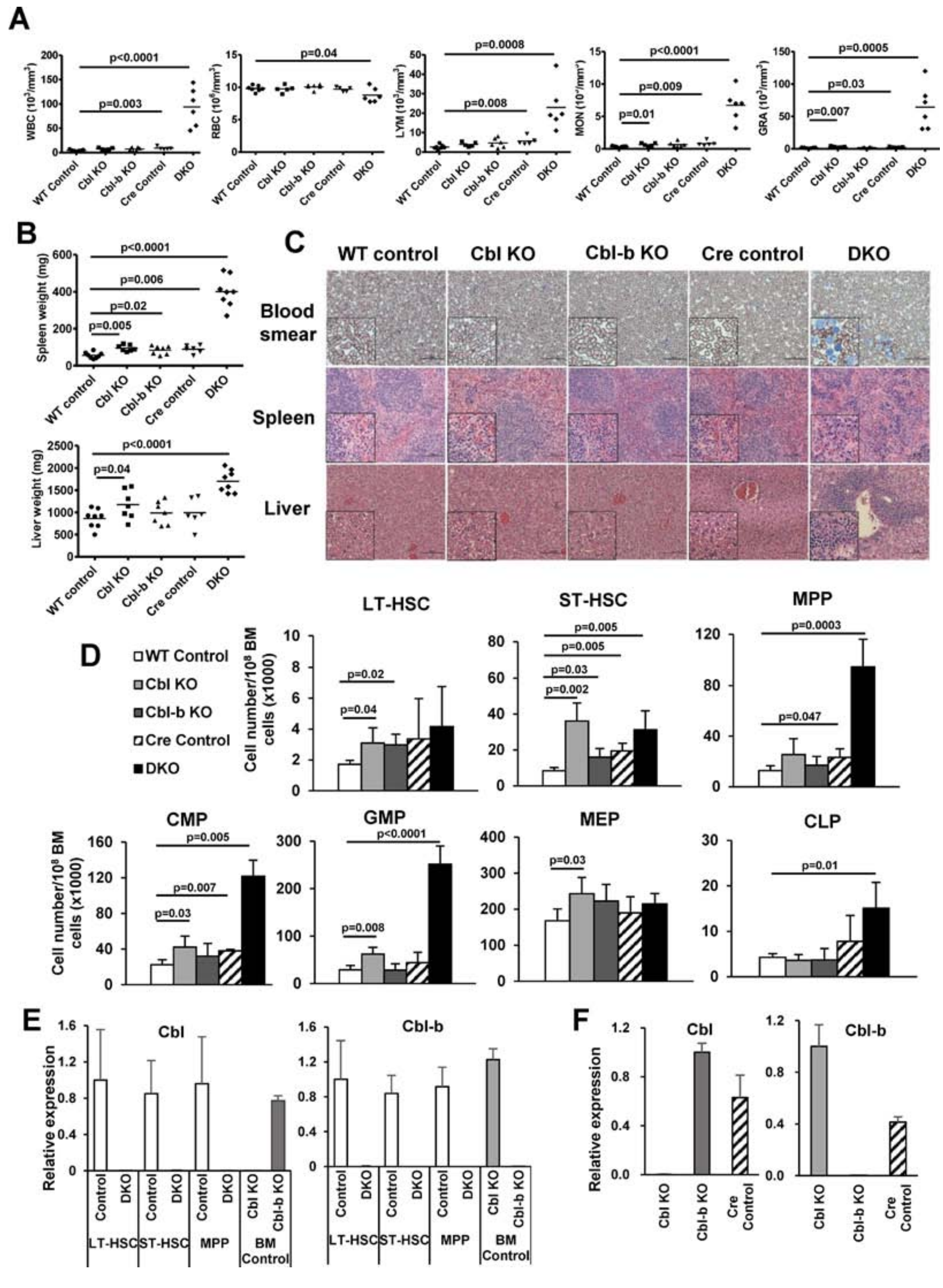

Supplementary Figure S1: Myeloproliferative disease (MPD) is uniquely observed in Cbl/Cbl-b DKO mice. **A.** Peripheral blood cell counts on mice of the indicated genotypes. WBC, white blood cell; LYM, lymphocyte; MON, monocyte; GRA, granulocyte; RBC, red blood cell. **B.** Spleen and liver weights. Dots represent individual mice. **C.** Representative Wright-Giemsa stained peripheral blood smears, and H&E stained spleen and liver sections. Higher magnification insets highlight leukocytosis in peripheral blood, loss of splenic architecture and perivascular leukocytes in liver. Bars, 0.1 mm. **D.** FACS analysis of bone marrow cells from mice of the indicated genotypes for HSCs (LT-HSC, ST-HSC and MPP) and progenitors (CMP, GMP, MEP and CLP). Data show mean ± SD of at least 3 mice for each genotype (*$p < 0.05$). long-term HSCs (LT-HSC, CD34$^-$ FLT3$^-$ Lin$^-$ Sca-1$^+$ c-Kit$^+$); short-term HSCs (ST-HSC, CD34$^+$ FLT3$^-$ Lin$^-$ Sca-1$^+$ c-Kit$^+$) and multipotent progenitors (MPP, CD34$^+$ FLT3$^+$ Lin$^-$ Sca-1$^+$ c-Kit$^+$). **E.** mRNA expression levels of Cbl (left) and Cbl-b (right) were analyzed in FACS-sorted LT-HSC, ST-HSC and MPP populations of WT control or Cbl/Cbl-b DKO by quantitative real-time PCR. **F.** mRNA levels of Cbl (left) and Cbl-b (right) in bone marrow cells from Cre Control mice (MMTV-Cre, Cbl $^{flox/flox}$, Cbl-b $^{+/-}$) were analyzed by quantitative RT-PCR. Cbl-KO and Cbl-b-KO bone marrow cells was used as internal controls. mRNA level was normalized using GAPDH. Data are pooled from two independent experiments and shown as mean ± SD.

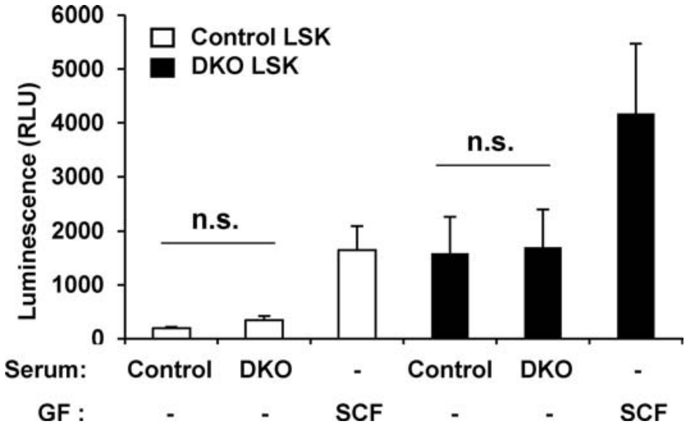

Supplementary Figure S2: WT or DKO LSK was unaltered upon adding DKO or control mouse sera. FACS-sorted LSK cells from control or Cbl/Cbl-b DKO mice were cultured in the presence of 10% serum derived from control or DKO mice. Cell proliferation was assayed using TiterGlo assay 3 days later. A representative experiment of three independent experiments is shown; 5 replicates in each experiment.

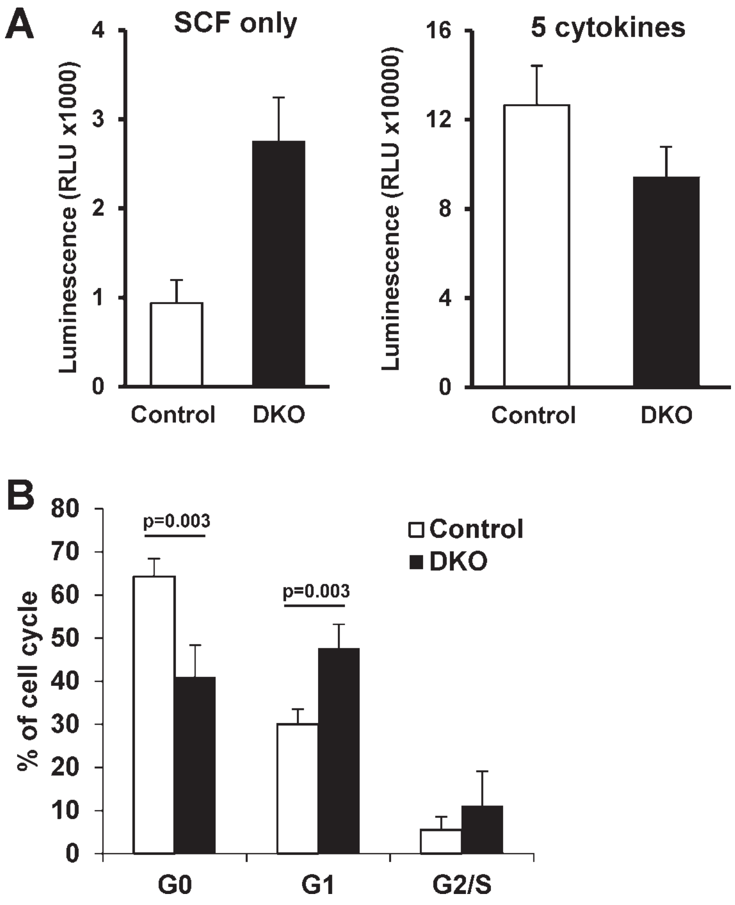

Supplementary Figure S3: Reduced quiescent HSC in DKO mice. A. FACS-sorted LSK cells were cultured for 3 days at 50 cells per well in 96-well plate with SCF (50 ng/ml) only (left) or 5 cytokines (right) including 50 ng/ml SCF, 20 ng/ml TPO, 10 ng/ml FLT3L, 10 ng/ml IL-3 and 10 ng/ml IL-6. Cells proliferation was evaluated by TiterGlo assay. A representative experiment of two independent experiments is shown; at least 3 replicates in each experiment. **B.** Cell cycle analysis of $CD48^-$ $CD150^+$ LSKs using Ki-67 and Hoechst staining. Data from at least three experiments are pooled and shown as mean ± SD. *$p < 0.05$.

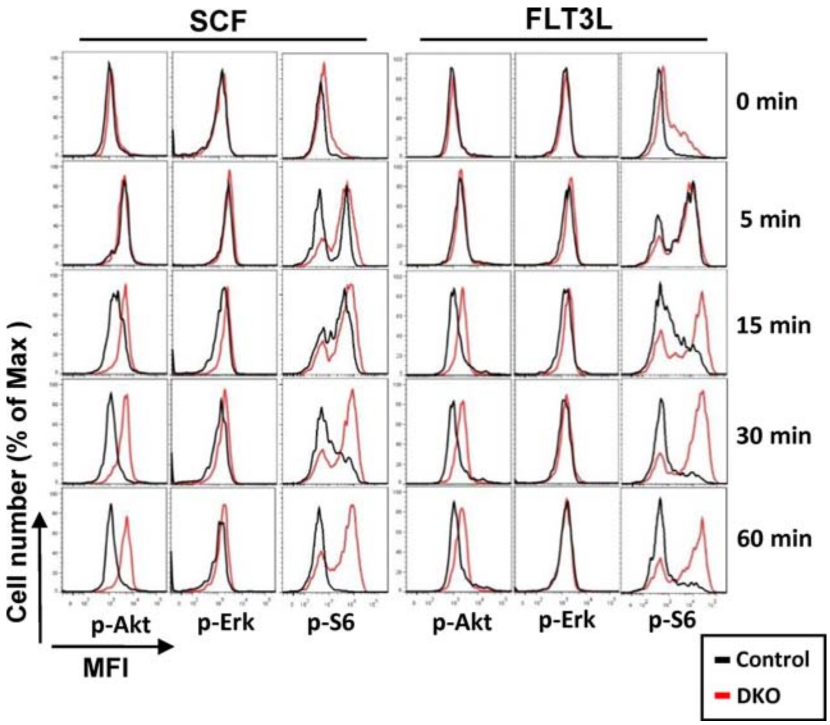

Supplementary Figure S4: Loss of Cbl and Cbl-b enhances c-Kit and FLT3 signaling in HSCs. Levels of p-Akt, p-Erk and p-S6 in unstimulated vs. ligand-stimulated LSK cells over time were analyzed by FACS (Phospho-FLOW). A representative FACS plot is shown.

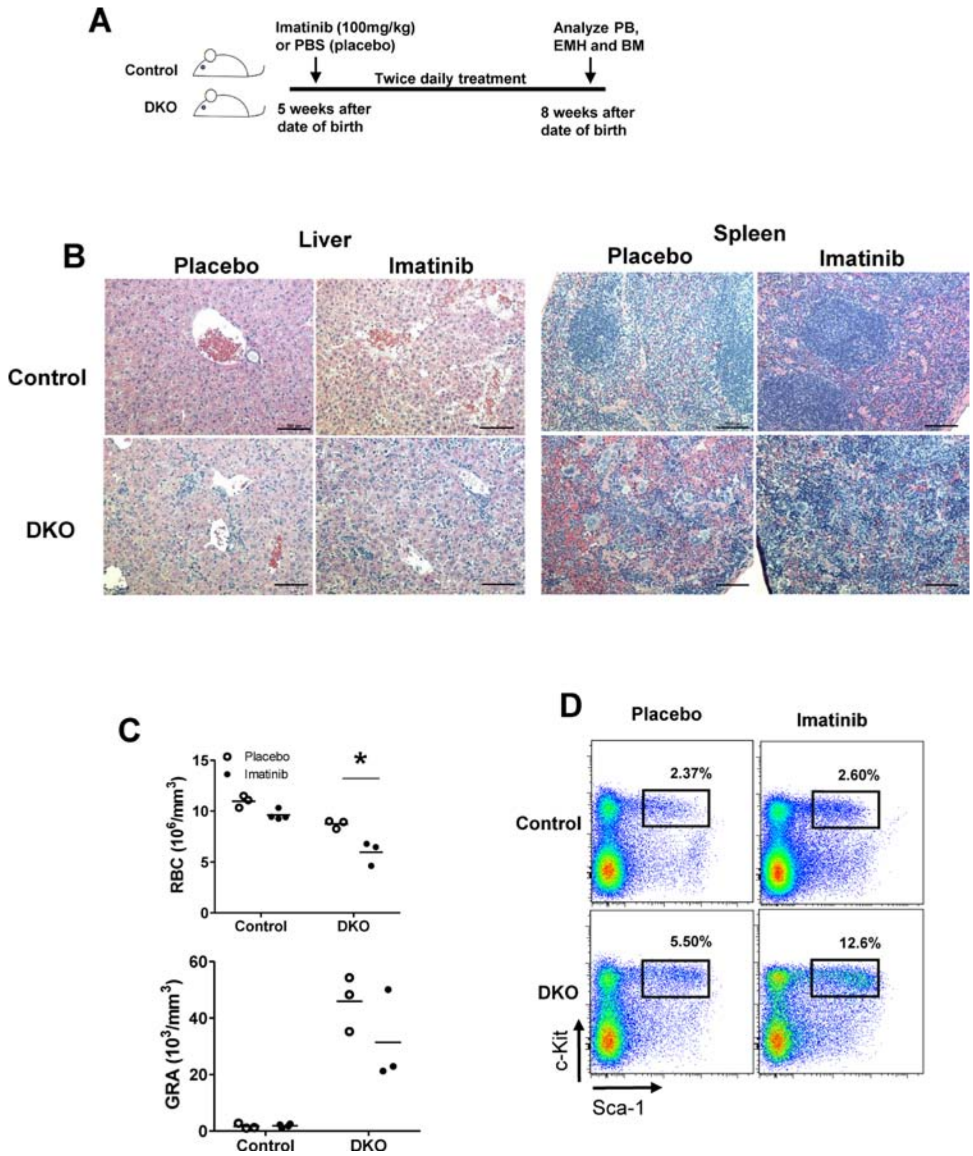

Supplementary Figure S5: Imatinib *in vivo* treatment. **A.** Experimental design. **B.** Representative H&E stained spleen and liver sections. Bars, 0.1 mm. **C.** Peripheral blood cell counts (RBC and GRA) on mice with indicated treatment. **D.** Bone marrow analysis. Representative FACS plots are shown.

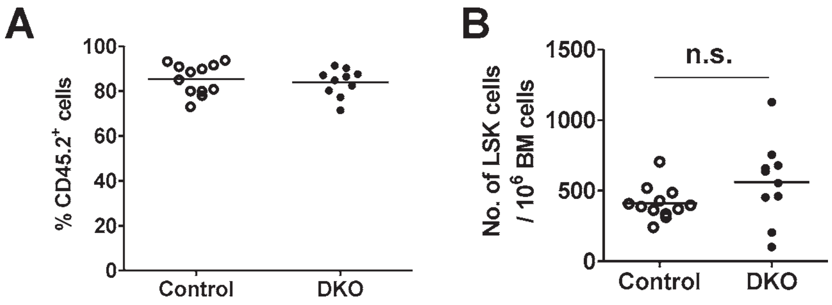

Supplementary Figure S6: Cbl/Cbl-b DKO HSCs display impaired reconstitution ability. A. Peripheral blood chimerism of recipient mice with >70% donor cell reconstitution. **B.** Donor LSK cells counts in BM of recipients shown in Fig 5C and 5D. Data are pooled from three independent experiments.

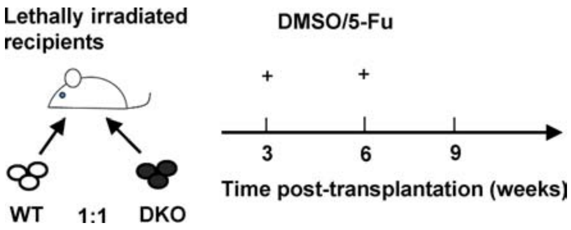

Supplementary Figure S7: 5-FU promote DKO HSC exhaustion. Experimental design.

Supplementary Table S1. Genotypes of mice used in this paper

Strain designation	Genotype
WT Control	Cbl $^{WT/WT}$ Cbl-b $^{WT/WT}$ or Cbl $^{flox/flox}$ Cbl-b $^{WT/del}$
Cbl KO	Cbl $^{del/del}$
Cbl-b KO	Cbl $^{flox/flox}$, Cbl-b $^{del/del}$
Cre Control	MMTV-Cre $^{Tg/0}$ Cbl $^{flox/flox}$, Cbl-b $^{WT/del}$
Cbl/Cbl/-b DKO	MMTV-Cre $^{Tg/0}$ Cbl $^{flox/flox}$, Cbl-b $^{del/del}$

Supplementary Table S2. Primers used for genotyping

Target allele	Forward primer 5′-3′	Reverse primer 5′-3′
**Cbl WT**	AAGTTCCAAGCCTAGCCAGATATGTGTGTG	TCCCCTCCCCTTCCCATGTTTT TAATAGACTC
**Cbl Deletion**	TGGCTGGACGTAAACTCCTCTTCAGACCTAATAAC	TCCCCTCCCCTTCCCATGTTTT TAATAGACTC
**Cbl-b**	CCCAGCAAAAGTAGCCAATG	CTTGCAAAAAGGACTAAGATTC
**MMTV-Cre**	GCGGTCTGGCAGTAAAAACTATC	GTGAAACAGCATTGCTGTCACTT

Supplementary Table S3. Primers used for quantitative real-time PCR

Target	Forward 5′-3′	Reverse 5′-3′
**Cbl**	AGCTGATGCTGCCGAATTT	TTGCAGGTCAGATCAATAGTGG
**Cbl-b**	GGAGCTTTTTGCACGGACTA	TGCATCCTGAATAGCATCAA
**p57**	GCGCAAACGTCTGAGATGAGT	AGAGTTCTTCCATCGTCCGCT
**p27**	AGTGTCCAGGGATGAGGAAGCGAC	TTCTTGGGCGTCTGCTCCACAGTG
**p21**	CCGCTGGAGGGCAACTTCGT	TTTCGGCCCTGAGATGTTCC
**GAPDH**	CCTGGAGAAACCTGCCAAGTATG	AGAGTGGGAGTTGCTGTTGAAGT
